# Health professions education and globalization: a call for reflexivity

**Published:** 2016-12-05

**Authors:** Brian Hodges

**Affiliations:** 1 University Health Network, University of Toronto

We seem to be subject to an ever more strident globalization discourse. Leaders, political parties, countries, and international organizations increasingly work to create an identity that is either strongly aligned with or against globalization. In the context of such discursive ferment, health professions education cannot escape the conversation. And yet, a read of our literature reflects more confusion than clarity. Only seven years ago, my colleagues and I conducted a review of the literature on globalization in medical education and found next to nothing to report.^1^ Concerns abounded, of course: the nefarious effects of students going abroad unsupervised, exporting culturally inappropriate teaching, and poaching health professionals from low income countries under the guise of international education. But those concerns were largely imported from other fields and sprinkled atop a medical education field that had, as yet, scarcely engaged with the issues.^2^

Not yet a decade later, medical educators are turning the full force of their intellect to what it means, and what it does, to engage globally. Good quality research on globalization is now available. Martimianakis and Hafferty’s careful empirical work illustrates how easily students and teachers become conflicted about the way globalization is presented in the classroom – mixing discourses of universal competence, cultural specificity, and advocacy.^3^ Paul et al. similarly showed up inconsistencies in Canada’s national policies and practices that simultaneously convey to internationally trained health professionals that they are welcome, but also that they are potentially incompetent; that they are a needed part of the labour force, but also that they upend the domestic job market.^4^ Ho et al. challenged the generalization of competencies^5^ and Frambach et al. questioned the transferability of pedagogical methods.^6^

It is not a question of *if* health sciences schools, their faculty and students should participate in globalization. Today’s questions are, rather, when they do engage internationally, what is their intention, how do they partner, and what are the consequences? To date, schools, faculty, and students have been passive with regard to these questions - perhaps because of naiveté but also, I believe, because of a failure to consider the ethical dimension of international activity.

A novel approach to countering that passivity comes from the University of Washington. In the spirit of the “plastic pocket card” that is ubiquitous in medical education, Mathew Sparke and colleagues created a card that illustrates, not how to resuscitate a patient, or how to escalate a crisis, but how to reflect on the impact of international activities. For example, the card asks the user to consider if they might be involved in: tourism (a vacation at risk of objectifying poor countries), romanticism (over emphasizing what can be accomplished with volunteerism), parasitism (incurring costs to a low income host), careerism (cv burnishing), or exemptionalism (doing things one would not be permitted to do at home).^7^ The card puts ethical questions directly into the pocket of the faculty member or student.

Authors including Whitehead ask us to think hard about the geo-political dimensions in which our global activities are embedded.^8^ In an era when medical schools hold tightly to concepts of social responsibility, researchers like Whitehead are asking us to question the impact of our international engagements. A clamouring for international partnerships between institutions makes this work more urgent. For example, University of Toronto’s 13-year partnership with the University of Addis Ababa has stimulated a great deal of reflection, debate, and learning on both sides.^9^ From these experiences, I propose a sort of plastic pocket card for health sciences schools. It might include the following questions:

1. How does the project align with the social responsibility of your school and to the city/region/country/society in which it is located? How will the project affect the social responsibility of your partner’s school/city/region/country/society?
2. Does the project improve (or worsen) inequities in the access to health care by patients, or to education by students, in either place?
3. Is the program in line with health-human resource planning to build national capacity of all countries involved?
4. In developing the activities, is there robust participation by, and credit given, to all partners?
5. Does the project impose any healthcare or education standards, assumptions, or practices from an economically or culturally dominant country that do not fit with the cultural, economic, healthcare, or education needs of the partner(s)?

The many interesting articles in this special edition grapple with these and other challenges. Canada has stood at the nexus of international travel, trade, and engagement since the first Europeans imposed themselves onto the land of North America’s indigenous peoples. The jostling of cultures and civilizations around the world continues apace. As the authors of these papers illustrate, health professions education is at its best when we reflect on the meaning, ethics, and implications of our programs and practices.
